# Catalpol Inhibits Ischemia-Induced Premyelinating Oligodendrocyte Damage through Regulation of Intercellular Calcium Homeostasis via Na^+^/Ca^2+^ Exchanger 3

**DOI:** 10.3390/ijms19071925

**Published:** 2018-06-30

**Authors:** Qiyan Cai, Teng Ma, Yanping Tian, Chengren Li, Hongli Li

**Affiliations:** Chongqing Key Laboratory of Neurobiology, Department of Histology and Embryology, College of Basic Medicine, Army Medical University (Third Military Medical University), Chongqing 400038, China; fengcai1112@126.com (Q.C.); matt0119@163.com (T.M.); tianyp1981@163.com (Y.T.); lichengren@sohu.com (C.L.)

**Keywords:** catalpol, premyelinating oligodendrocytes, ischemia, calcium homeostasis, Na^+^/Ca^2+^ exchanger 3

## Abstract

The heightened vulnerability of premyelinating oligodendrocytes (PreOLs) in response to hypoxia–ischemia may contribute to perinatal white matter injury and subsequent neurobehavioral dysfunction. Intracellular Ca^2+^ overload is considered a crucial mechanism predisposing PreOLs to ischemic injury. We previously reported that catalpol, an iridoid glycoside extracted from Rehmannia root, inhibits intracellular Ca^2+^ overload of PreOLs in an in vitro ischemia model. However, the exact underlying mechanisms remain elusive. In the present study, we aimed to investigate the protective effects of catalpol on PreOLs and to explore the underlying mechanisms involved in the modulation of intracellular Ca^2+^ homeostasis. Postnatal day 2 (P2) Sprague-Dawley (SD) rats subjected to bilateral common carotid artery ligation followed by exposure to 8% oxygen for 10 min were used as a rat model of neonatal hypoxia–ischemia. We found that catalpol significantly improved behavioral functions and prevented PreOL loss and myelination deficit after hypoxia–ischemia. Our in vitro studies also confirmed the direct effects of catalpol on oxygen-glucose deprivation (OGD)-induced cell death and arrested maturation of PreOLs. Moreover, we demonstrated that catalpol significantly inhibited intracellular Ca^2+^ overload and promoted the expression of Na^+^/Ca^2+^ exchanger 3 (NCX3). Finally, we found that catalpol significantly reduced mitochondrial damage and subsequent extracellular signal-regulated kinase 1/2 (ERK1/2) and poly-ADP-ribose polymerase-1 (PARP-1) activation. Treatment with NCX3-preferring inhibitor 2-[2-[4-(4-nitrobenzyloxy)phenyl]ethyl]isothiourea (KB-R7943) significantly reversed the protective effects of catalpol on PreOLs under OGD. Overall, our data suggest that catalpol protects PreOLs from ischemic injury through regulation of intercellular Ca^2+^ homeostasis via upregulation of NCX3 activity.

## 1. Introduction

Periventricular white matter injury is a predominant form of perinatal brain injury and hypoxia–ischemia is thought to be the leading cause [1]. During the high-risk period for perinatal white matter injury, white matter is predominantly populated by developing oligodendrocytes (OLs), termed premyelinating oligodendrocytes (PreOLs) [2]. Abundant evidence shows that PreOLs are highly vulnerable to hypoxia-ischemia, whereas earlier- and later-stage cells are markedly more resistant [3]. The heightened vulnerability of PreOLs in response to hypoxia–ischemia may contribute to arrested OL maturation and subsequent myelination failure, which finally impair white matter function [4].

Intracellular Ca^2+^ is an important second messenger involved in signal transduction. The balance between Ca^2+^ influx and efflux is critical for cell migration, differentiation, myelination, and survival in OL lineage cells [5,6,7]. When intracellular Ca^2+^ homeostasis is perturbed by ischemia, an elevation in cytosolic Ca^2+^ concentration occurs, which is attributable to Ca^2+^ influx through the plasma membrane and intracellular stores [7]. This massive Ca^2+^ influx causes excessive activation of Ca^2+^-dependent signaling pathways, leading to the damage or death of OL lineage cells [7,8]. Recent evidence has suggested that dysfunction of plasma membrane Ca^2+^ extrusion systems may also contribute to sustained intracellular Ca^2+^ overload following ischemic brain damage by blocking the extrusion of excess cytosolic Ca^2+^ [9,10].

The Na^+^/Ca^2+^ exchanger (NCX) isoforms NCX1, NCX2, and NCX3 mediate Na^+^ and Ca^2+^ fluxes in a bidirectional way across the plasma membrane and constitute the major plasma membrane Ca^2+^ extrusion system widely distributed in the brain [11]. Accumulating data suggest that reduced NCX activity following aberrant transcription or proteolytic cleavage of these exchangers may worsen ischemic brain damage by dysregulating Na^+^ and Ca^2+^ homeostasis [12,13,14]. NCX3, recently identified as a myelin membrane component, plays important roles in the regulation of intracellular Ca^2+^ concentrations during OL maturation [15]. *NCX3* gene ablation impaired OL precursor response and increased susceptibility to experimental autoimmune encephalomyelitis [16]. Our recent study showed that lead poisoning disturbs PreOL differentiation and viability in association with decreased expression of NCX3, thus inducing intracellular Ca^2+^ overload [17]. These observations suggest that approaches to protect against loss of NCX3 function may be useful to counteract potentially harmful Ca^2+^ loads leading to PreOL demise under ischemia.

Catalpol, an iridoid glycoside separated from Rehmannia root, produces superior neuroprotective effects in both in vitro and in vivo studies [18,19]. Catalpol can protect primary cultured astrocytes and rat pheochromocytoma cells against ischemic injury [20,21]. Catalpol has also been demonstrated to promote OL survival and myelin repair in a rat chronic cerebral hypoperfusion model [22,23]. Catalpol attenuates mitochondrial dysfunction by reducing intracellular Ca^2+^ levels in mesencephalic neuron-astrocyte cultures damaged by 1-methyl-4-phenyl-1,2,3,6-tetrahydropyridine (MPTP) [18]. Recently, we demonstrated that catalpol inhibits oxygen-glucose deprivation (OGD)-induced intracellular Ca^2+^ overload in PreOLs [24]. However, the underlying mechanisms by which catalpol regulates intracellular Ca^2+^ concentration remain elusive.

In the present study, we used a rat model of neonatal hypoxia–ischemia and an in vitro ischemia model to investigate the protective effects of catalpol on PreOLs and to explore the underlying mechanisms involved in the modulation of intracellular Ca^2+^ homeostasis. We found that catalpol significantly improved the behavioral disorders of rats and provided potent neuroprotection to PreOLs after hypoxia-ischemia. We also demonstrated that catalpol can inhibit the death and arrested maturation of PreOLs by inhibiting intracellular Ca^2+^ overload, mitochondrial damage, and subsequent extracellular signal-regulated kinase 1/2 (ERK1/2) activation under OGD. Upregulated NCX3 activity was shown to contribute to these effects.

## 2. Results

### 2.1. Catalpol Improves Motor and Memory Deficits of Rats after Neonatal Hypoxia–Ischemia

**Figure 1:**
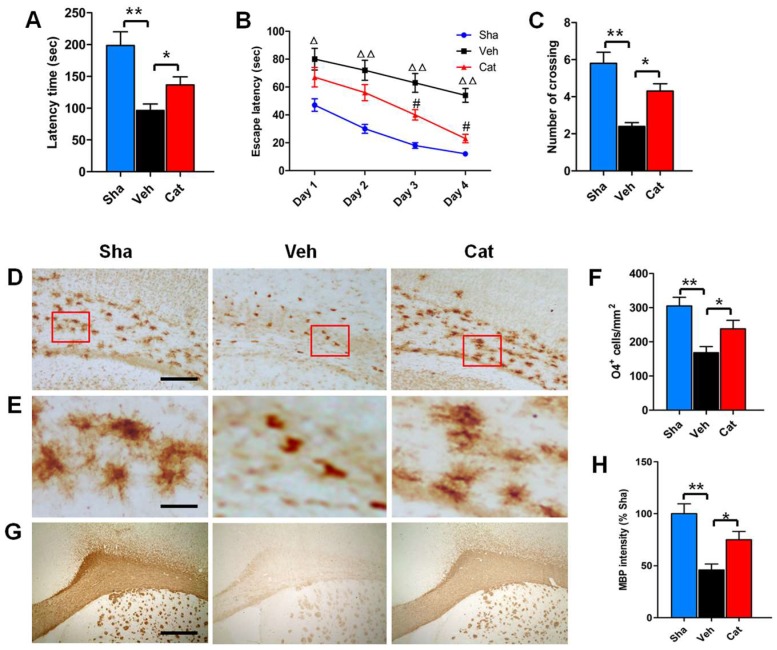
Effects of catalpol on behavioral outcome, premyelinating oligodendrocytes (PreOL) damage, and myelination in a rat model of neonatal hypoxia–ischemia. (**A**) Rotarod test of the latency time for remaining on the cylinder in the sham-control (Sha), vehicle-treated (Veh), and catalpol-treated (Cat) groups. (**B**) Morris water maze test of the escape latency to find the platform in the Sha, Veh, and Cat groups. (**C**) Number of crossings of the previous platform location in the Morris water maze test in the Sha, Veh, and Cat groups. (**D**) Representative O4 staining in the corpus callosum of the Sha, Veh, and Cat groups. Bar = 100 μm. (**E**) High magnification images of O4 staining in the corpus callosum of the Sha, Veh, and Cat groups. Bar = 25 μm. (**F**) Quantification of O4-positive cells in the corpus callosum of the Sha, Veh, and Cat groups. (**G**) Representative myelin basic protein (MBP) staining in the corpus callosum of the Sha, Veh, and Cat groups. Bar = 500 μm. (**H**) Quantification of the optical density of MBP staining in the corpus callosum of the Sha, Veh, and Cat groups. Data are shown as means ± SEM (*n* = 6 in each group). * *p* < 0.05, ** *p* < 0.01 vs. indicated group; ^△^*p* < 0.05, ^△△^*p* < 0.01 vs. Sha; ^#^*p* < 0.05 vs. Veh.

Motor and memory deficits are common behavioral and cognitive impairments of rats subjected to neonatal hypoxia-ischemia brain injury [25,26]. To examine the effects of catalpol on motor coordination and balance, the Rotarod test was performed on rats from P23 to P25. The results of Rotarod test showed that the average latency time that the vehicle-treated (Veh) group remained on the cylinder was decreased to 48.47% of that of the sham-control (Sha) group (*p* < 0.01), demonstrating the motor dysfunction caused by hypoxia–ischemia. Treatment with 5 mg/kg catalpol increased the average latency time of remaining on the cylinder to 141.74% of the Veh group (*p* < 0.05) (Figure 1A), indicating improved motor coordination. We further used the Morris water maze test to measure the effects of catalpol on spatial learning and memory of rats. During the training trials, the escape latency (time to reach the platform) was recorded every day to evaluate spatial learning. It was found that the escape latency was significantly longer in the Veh group than that in the Sha group from P26 to P29 (day 1, *p* < 0.05; days 2–4, *p* < 0.01). Catalpol treatment significantly reduced the escape latency of rats to find the platform (days 3 and 4, *p* < 0.05) (Figure 1B). After completion of training trials, the spatial memory of rats was measured in the probe test on P30. The platform was removed and the number of crossings of the previous platform location was recorded. We found that the number of crossings in the Veh group was about 2.4 times, which showed a significant difference to the Sha group (*p* < 0.01). However, the catalpol-treated (Cat) group had about 4.3 times crossing frequency, with a significant difference compared with the Veh group (*p* < 0.05) (Figure 1C). Our data suggested the motor and memory deficits induced by neonatal hypoxia–ischemia can be reversed by catalpol.

### 2.2. Catalpol Prevents PreOL Damage and Myelination Deficit after Hypoxia–Ischemia

To determine the effects of catalpol on perinatal white matter injury, we used immunohistochemisty to analyze the PreOL damage and myelination deficit of white matter subjected to neonatal hypoxia–ischemia. O4 (a marker of PreOLs) immunostaining was abundant in the white matter of the Sha group on P6. The O4-positive cells were larger and had multiple branches in the Sha group. Hypoxia–ischemia resulted in severe loss of O4 immunostaining in the white matter area, with a significant difference compared with the Sha group (*p* < 0.01). Most of the O4-positive cells in the Veh group displayed degenerative features with few processes and an increase in cell density. Catalpol treatment not only significantly increased the number of O4-positive cells in the white matter (*p* < 0.05), but also markedly attenuated the degenerative profile of PreOLs under hypoxia–ischemia (Figure 1D–F). On P30, myelin basic protein (MBP) (a major constituent of the myelin sheath) immunostaining was clearly observed in the white matter of the Sha group. However, MBP immunostaining was very weak in the white matter of the Veh group. Statistical analysis showed that the mean optical density of MBP staining in the white matter of the Veh group was significantly decreased compared with the Sha group (*p* < 0.01). Catalpol treatment significantly improved myelination, as demonstrated by the elevated mean optical density of MBP staining (*p* < 0.05) (Figure 1G,H). These results demonstrate that catalpol prevents white matter damage induced by neonatal hypoxia–ischemia.

### 2.3. Catalpol Improves Survival and Restores Maturation of PreOLs under OGD

**Figure 2:**
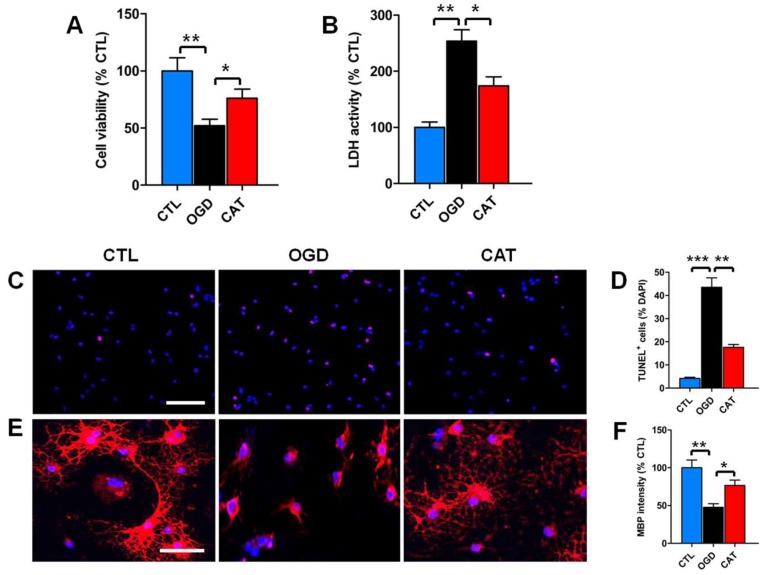
Effects of catalpol on PreOL survival and maturation under oxygen-glucose deprivation (OGD). (**A**) Quantification of cell viability as measured by the 3-(4,5-dimethylthiazol-2-yl)-2,5-diphenyltetrazolium bromide (MTT) assay in the control (CTL), OGD-treated (OGD), and catalpol-treated (CAT) groups. (**B**) Quantification of lactate dehydrogenase (LDH) activity in the CTL, OGD, and CAT groups. (**C**) Representative terminal deoxynucleotidyl transferase-mediated dUTP-biotin nick end labeling assay (TUNEL) staining (red) in the CTL, OGD, and CAT groups. Cell nuclei were counterstained with 4′,6′-diamidino-2-phenylindole (DAPI) (blue). Bar = 50 μm. (**D**) Quantification of TUNEL-positive cells in the CTL, OGD, and CAT groups. (**E**) Representative MBP staining (red) in the CTL, OGD, and CAT groups. Cell nuclei were counterstained with DAPI (blue). Bar = 25 μm. (**F**) Quantification of the optical density of MBP staining in the CTL, OGD, and CAT groups. Data are shown as means ± SEM (*n* = 6 in each group). * *p* < 0.05; ** *p* < 0.01; *** *p* < 0.001.

To identify the protective effects of catalpol treatment on PreOLs subjected to OGD, 3-(4,5-dimethylthiazol-2-yl)-2,5-diphenyltetrazolium bromide (MTT), and cytosolic lactate dehydrogenase (LDH) assays were used to measure cell viability and cytotoxicity after 3 days of differentiation. The results of the MTT assays revealed that OGD had an apparent inhibitory effect on cell viability compared to the control conditions (*p* < 0.01). By contrast, treatment with catalpol significantly decreased the inhibitory effect of OGD on cell viability (*p* < 0.05) (Figure 2A). These findings were further verified by LDH assay. The release of LDH was significantly increased in cultures exposed to OGD alone compared to the control (CTL) group (*p* < 0.01), and this increase was markedly inhibited by treatment with catalpol (*p* < 0.05) (Figure 2B).

To further investigate the effect of catalpol on OGD-induced apoptosis, terminal deoxynucleotidyl transferase-mediated dUTP-biotin nick end labeling assay (TUNEL) staining was performed to quantitatively estimate the number of apoptotic cells. Very few TUNEL-positive apoptotic cells were detected in the CTL group. However, the number of apoptotic cells was significantly higher upon OGD stimulation than in the control conditions (*p* < 0.001). This increased number of apoptotic cells was markedly reduced by administration of catalpol (*p* < 0.01) (Figure 2C,D). These data collectively suggest that catalpol significantly improves the survival of PreOLs after OGD.

To define the function of catalpol in the maturation of PreOLs after OGD, immunofluorescence staining was performed to examine the expression level of MBP in PreOL cultures on day 6 of differentiation. The cells in the CTL group exhibited elaborate networks of cellular processes. By contrast, the cells in the OGD-treated (OGD) group appeared more immature and exhibited a simple morphology with few processes. Statistical analysis showed that the fluorescence intensity of MBP staining in the OGD group was significantly decreased relative to the CTL group (*p* < 0.01). However, delayed differentiation and maturation were less evident in the catalpol-treated (CAT) group, as shown by the presence of more complex, multibranched processes and the increased fluorescence intensity of MBP staining (*p* < 0.05) (Figure 2E,F). These results suggest that catalpol restores the developmental capacity of PreOLs to mature under OGD.

### 2.4. Catalpol Inhibits Intracellular Calcium Elevation of PreOLs under OGD

To determine the effect of catalpol on intracellular Ca^2+^ levels, which influence the viability and maturation of oligodendrocyte progenitor cells (OPCs) when elevated [17,27], we utilized the Ca^2+^-specific fluorescent probe Fluo-3/AM to measure the alterations of Ca^2+^ levels at the resting condition in PreOLs after OGD. In order to observe the regional difference of PreOLs in the Ca^2+^ response, we transformed the time-lapse Ca^2+^ imaging into pseudocolor changes and 3D surface plots. 

**Figure 3:**
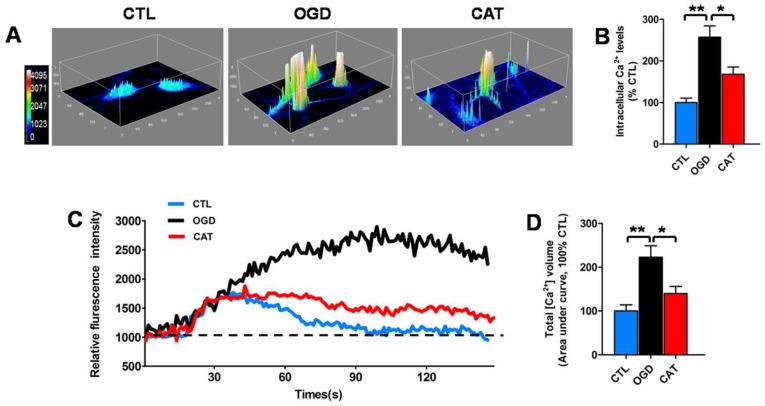
Effects of catalpol on intracellular Ca^2+^ levels in PreOLs under OGD. (**A**) Representative 3D images of Fluo-3 staining in the CTL, OGD, and CAT groups. Intracellular Ca^2+^ levels (Fluo-3 staining) at the resting condition are indicated with a pseudocolor range (left side, color scale 0–4095). (**B**) Quantification of the fluorescence intensity of Fluo-3 in the CTL, OGD, and CAT groups. (**C**) Representative Ca^2+^ response traces under high-glucose stimulation in the CTL, OGD, and CAT groups. (**D**) Quantification of total volume of intracellular Ca^2+^ under high-glucose stimulation in the CTL, OGD, and CAT groups. The area under the curve represents the total volume of intracellular Ca^2+^. Six separate experiments were conducted and 15–30 cells were recorded for each experiment. Data are shown as means ± SEM. * *p* < 0.05; ** *p* < 0.01.

In the CTL group, a weak Ca^2+^ response was only found in the somal regions of PreOLs. By contrast, the Ca^2+^ response was strong in both the soma and processes of PreOLs subjected to OGD. Intracellular Ca^2+^ concentrations were significantly elevated in PreOLs after OGD injury, as indicated by the markedly elevated fluorescence signals of Fluo-3 compared to the CTL group (*p* < 0.01). In the CAT group, Ca^2+^ response was reduced in both the soma and processes. Intracellular Ca^2+^ concentrations were significantly decreased by catalpol treatment, as demonstrated by the decreased fluorescence signals of Fluo-3 compared with the OGD group (*p* < 0.05) (Figure 3A,B).

In addition, we also monitored the real-time changes in the intracellular Ca^2+^ response under 30 mM high-glucose stimulation. In the CTL group, intracellular Ca^2+^ concentrations showed a transient elevation and then rapidly returned to basal levels following high-glucose stimulation. By contrast, in PreOLs subjected to OGD, intracellular Ca^2+^ concentrations rose steadily throughout the period. The total volume of intracellular Ca^2+^ in the OGD group was strongly increased, as shown by the markedly elevated area under the curve compared to the CTL group (*p* < 0.01). Treatment with catalpol improved the recovery of Ca^2+^ concentrations toward to basal levels under high-glucose stimulation. The total volume of intracellular Ca^2+^ was significantly decreased following catalpol treatment, as demonstrated by the decreased area under curve compared with the OGD group (*p* < 0.05) (Figure 3C,D). These data demonstrated that catalpol protects PreOLs against OGD-induced injury by normalizing intracellular Ca^2+^ levels.

### 2.5. Catalpol Enhances NCX3 Expression in PreOLs under OGD

**Figure 4:**
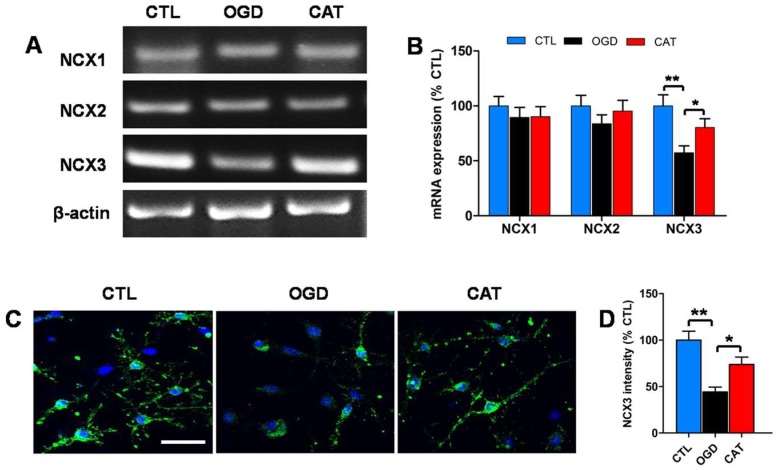
Effects of catalpol on Na^+^/Ca^2+^ exchanger 3 (*NCX3*) expression in PreOLs under OGD. (**A**) RT-PCR to probe Na^+^/Ca^2+^ exchanger 1 (*NCX1*), Na^+^/Ca^2+^ exchanger 2 (*NCX2*), and *NCX3* mRNA levels in the CTL, OGD, and CAT groups. *β-actin* was used as control. (**B**) Quantification of *NCX1*, *NCX2*, and *NCX3* mRNA levels from the RT-PCR analyses. (**C**) Representative NCX3 staining (green) in the CTL, OGD, and CAT groups. Cell nuclei were counterstained with DAPI (blue). Bar = 25 μm. (**D**) Quantification of the optical density of NCX3 staining in the CTL, OGD, and CAT groups. Data are shown as means ± SEM (*n* = 6 in each group). * *p* < 0.05; ** *p* < 0.01.

To determine whether catalpol inhibits intracellular Ca^2+^ elevation by regulating the functions of NCXs, RT-PCR was performed to analyze the mRNA levels of *NCX1*, *NCX2*, and *NCX3* in PreOLs on day 3 of differentiation. OGD injury or catalpol treatment had no significant effect on the expression levels of *NCX1* and *NCX2* mRNA. The mRNA level of *NCX3* was significantly decreased in response to OGD (*p* < 0.01), and this decrease was effectively prevented by catalpol treatment (*p* < 0.05) (Figure 4A,B). Immunofluorescence staining was further used to investigate the protein expression of NCX3. Similarly, OGD caused a conspicuous reduction in NCX3 protein expression compared to the CTL group (*p* < 0.01). Administration of catalpol markedly abrogated the decreased expression of NCX3 relative to the OGD group (*p* < 0.05) (Figure 4C,D). Therefore, our results suggest that catalpol inhibits intracellular Ca^2+^ elevation likely by modulating NCX3 in PreOLs induced by OGD.

### 2.6. Catalpol Protects PreOLs against OGD by Upregulating NCX3

To further define the contribution of NCX3 to catalpol-mediated protection, we evaluated changes in intracellular Ca^2+^ levels, LDH release, cell apoptosis, and MBP expression in PreOLs in the presence of NCX3-preferring inhibitor 2-[2-[4-(4-nitrobenzyloxy)phenyl]ethyl]isothiourea (KB-R7943) and catalpol. KB-R7943 was reported to be more selective to block NCX3 than NCX1 and NCX2 [28]. In previous tests, we found that KB-R7943 and NCX3 antibody have similar effects on Ca^2+^ response in PreOLs under OGD and high-glucose stimulation in the presence of catalpol (Figure S1). Thus, KB-R7943 was used as a NCX3-preferring inhibitor in the subsequent experiments.

**Figure 5:**
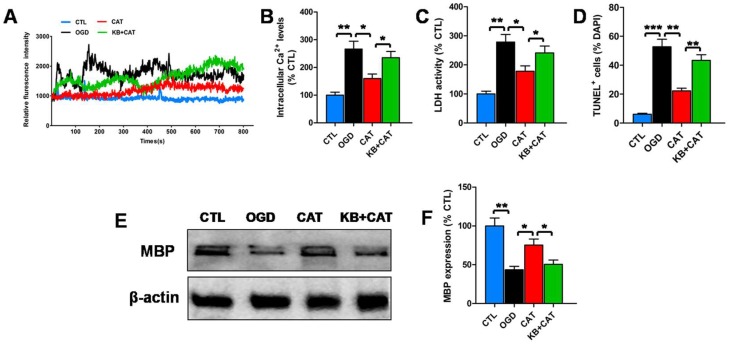
Effects of catalpol on intracellular Ca^2+^ levels, cytosolic LDH activity, cell survival, and maturation of PreOLs under OGD and 2-[2-[4-(4-nitrobenzyloxy)phenyl]ethyl]isothiourea (KB-R7943) treatment. (**A**) Representative Ca^2+^ response traces under resting condition in the CTL, OGD, CAT, and KB-R7943+catalpol-treated group (KB+CAT) groups. (**B**) Quantification of intracellular Ca^2+^ levels at the resting condition measured by Fluo-3 staining in the CTL, OGD, CAT, and KB+CAT groups. (**C**) Quantification of LDH activity in the CTL, OGD, CAT, and KB+CAT groups. (**D**) Quantification of TUNEL-positive cells in the CTL, OGD, CAT, and KB+CAT groups. (**E**) Western blot probing for MBP in the CTL, OGD, CAT, and KB+CAT groups. β-actin was used as a loading control. The blot shown is representative of six independent experiments. (**F**) Quantification of MBP protein expression levels from the Western blot analyses. Data are shown as means ± SEM (*n* = 6 in each group). * *p* < 0.05; ** *p* < 0.01; *** *p* < 0.001.

Intracellular Ca^2+^ levels at the resting condition were significantly increased in OGD-injured PreOLs compared to normal PreOLs (*p* < 0.01), and catalpol treatment markedly inhibited the elevation of intracellular Ca^2+^ levels (*p* < 0.05). However, this inhibitory effect of catalpol on Ca^2+^ overload was significantly prevented by co-application of KB-R7943 and catalpol (*p* < 0.05) (Figure 5A,B). LDH release after OGD was also significantly increased compared with the CTL group (*p* < 0.01). Catalpol markedly inhibited OGD-induced LDH release (*p* < 0.05), and this effect was significantly inhibited by applying KB-R7943 plus catalpol (*p* < 0.05) (Figure 5C). Similarly, the number of apoptotic PreOLs induced by OGD was significantly decreased in the CAT group (*p* < 0.01), and this decrease in apoptosis was significantly prevented by co-application of KB-R7943 and catalpol (*p* < 0.01) (Figure 5D). Western blot analysis showed a conspicuous reduction in MBP protein expression on day 6 of differentiation in the OGD group relative to the CTL group (*p* < 0.01). Catalpol markedly increased the expression of MBP compared to the OGD group (*p* < 0.05); however, application of KB-R7943 plus catalpol significantly inhibited this increase (*p* < 0.05) (Figure 5E,F). These data indicate that catalpol-mediated cytoprotection in PreOLs is correlated with NCX3 function.

### 2.7. Catalpol Reduces Mitochondrion-Mediated Activation of the ERK1/2 Pathway in PreOLs under OGD

**Figure 6:**
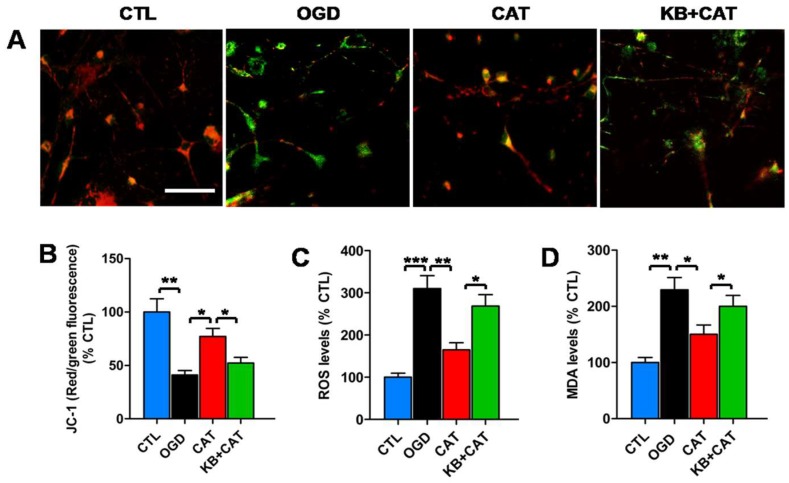
Effects of catalpol on mitochondrial damage in PreOLs under OGD and KB-R7943 treatment. (**A**) Representative JC-1 staining in the CTL, OGD, CAT, and KB+CAT groups. Bar = 25 μm. (**B**) Quantification of the red/green fluorescence ratio in the CTL, OGD, CAT, and KB+CAT groups. The ratio of red/green fluorescence intensity represents the mitochondrial membrane potential. (**C**) Quantification of reactive oxygen species (ROS) levels in the CTL, OGD, CAT, and KB+CAT groups. (**D**) Quantification of malondialdehyde (MDA) levels in the CTL, OGD, CAT, and KB+CAT groups. Data are shown as means ± SEM (*n* = 6 in each group). * *p* < 0.05; ** *p* < 0.01; *** *p* < 0.001.

Ca^2+^-sensitive signaling pathways are associated with mitochondrion-mediated activation of ERK1/2 [29]. To further investigate the downstream mechanism of the protective effects of catalpol in PreOLs, we examined the alteration of ERK1/2 activation, which has been implicated in OGD-induced mitochondrial damage in PreOLs [24]. OGD markedly decreased mitochondrial membrane potential compared to the CTL group, as shown by the reduced red fluorescence (*p* < 0.01). Catalpol treatment significantly relieved the loss of mitochondrial membrane potential after OGD injury, as demonstrated by the increase in the red fluorescence ratio (*p* < 0.05), and this effect of catalpol was significantly inhibited in the presence of KB-R7943 (*p* < 0.05) (Figure 6A,B). The results of the dichlorodihydrofluorescein diacetate (DCFH-DA) assay showed that PreOLs in the OGD group exhibited a robust increase in reactive oxygen species (ROS) levels compared with the CTL group (*p* < 0.001). These elevated ROS levels were markedly reduced following catalpol treatment (*p* < 0.01). However, in the KB-R7943+catalpol-treated group (KB+CAT) group, the levels of ROS were significantly increased compared to those in the CAT group (*p* < 0.05) (Figure 6C). Similar to ROS, the levels of malondialdehyde (MDA) were much higher in the PreOLs subjected to OGD than in those from the CTL group (*p* < 0.01), and treatment with catalpol significantly inhibited this elevation (*p* < 0.05). Following NCX3 inhibition using KB-R7943, the decreased MDA levels were markedly elevated relative to the CAT group (*p* < 0.05) (Figure 6D).

**Figure 7:**
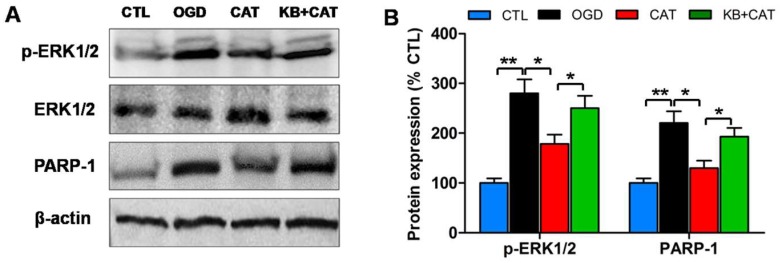
Effects of catalpol on the activation of the extracellular signal-regulated kinase 1/2 (ERK1/2) pathway in PreOLs under OGD and KB-R7943 treatment. (**A**) Western blot probing for p-ERK1/2 and poly-ADP-ribose polymerase-1 (PARP-1) from the CTL, OGD, CAT, and KB+CAT groups. Total ERK1/2 and β-actin were used as loading controls. The blot shown is representative of six independent experiments. (**B**) Quantification of p-ERK1/2 and PARP-1 protein expression levels from Western blot analyses. Data are shown as means ± SEM (*n* = 6 in each group). * *p* < 0.05; ** *p* < 0.01.

To correlate the ERK1/2 signaling pathway with catalpol-mediated protective effects on PreOLs, Western blot analysis was performed for p-ERK1/2 and poly-ADP-ribose polymerase-1 (PARP-1), an ERK1/2 target involved in cell death. The p-ERK1/2 protein levels were low in the CTL group, but they were substantially increased in the OGD group (*p* < 0.01), and these increases were effectively prevented by catalpol administration (*p* < 0.05). By contrast, co-application of KB-R7943 and catalpol reversed the effect of catalpol on ERK1/2 activation, as indicated by the elevated expression of the p-ERK1/2 protein, which differed significantly from that in the CAT group (*p* < 0.05). Similar to p-ERK1/2, PARP-1 protein levels were considerably higher in the OGD group than in the CTL group (*p* < 0.01), and catalpol significantly inhibited this elevation (*p* < 0.05). Following NCX3 inhibition using KB-R7943, PARP-1 protein levels were significantly increased compared to the CAT group (*p* < 0.05) (Figure 7A,B). These data indicate that the ERK1/2 signaling pathway may contribute to the effects of catalpol on PreOLs following OGD-induced intracellular Ca^2+^ overload.

## 3. Discussion

Hypoxia–ischemia causes white matter damage in preterm infants, which leads to widespread PreOL death and subsequent myelination failure [4,30]. Ischemia-induced intracellular Ca^2+^ overload is thought to be a principal mechanism predisposing PreOLs to injury, which triggers excessive activation of Ca^2+^-dependent signaling pathways [7,8]. In the present study, we found that catalpol remarkably improved both behavioral functions and myelination by preventing PreOL loss in a neonatal hypoxia-ischemia rat model. In addition, we used defined, stage-specific cultures to demonstrate that catalpol improved the survival and maturation of PreOLs under OGD toxicity. We further found that catalpol inhibited intracellular Ca^2+^ overload, mitochondrial damage, and the ERK1/2 signaling pathway by promoting the expression of NCX3 in PreOLs subjected to OGD.

Changes in intracellular Ca^2+^ play an important role in the damage or death of OL lineage cells, with excessive intracellular Ca^2+^ leading to overactivation of Ca^2+^-dependent signaling pathways, which are the final common mechanisms for cell damage or death [6,31]. Following intracellular Ca^2+^ overload, mitochondrial Ca^2+^ equilibrium is disrupted, which triggers mitochondrial dysfunction, oxidative stress, and eventually cell death [7,32,33]. Therefore, preventing intracellular Ca^2+^ overload may effectively inhibit cell damage. Catalpol was previously reported to reduce MPTP-induced intracellular Ca^2+^ elevation and mitochondrial dysfunction in mesencephalic neuron-astrocyte cultures [18]. Our study revealed that catalpol significantly blocked OGD-induced intracellular Ca^2+^ overload, mitochondrial damage, and ROS generation in PreOLs. These findings clearly demonstrate that catalpol has a role in mediating intracellular Ca^2+^ homoeostasis of PreOLs after ischemia and thereby decreases mitochondrial damage and cell death.

During cerebral ischemia, NCX can operate either in the forward mode to extrude Ca^2+^ or in the reverse mode to mediate Ca^2+^ influx resulting from the loss of Na^+^/K^+^-ATPase activity [13,34]. Alterations of the gene and protein expression of the NCX isoforms *NCX1*, *NCX2*, and *NCX3* are usually found in different brain regions damaged by ischemia [12,13,35]. Our experiments demonstrated that OGD induced a significant decrease in *NCX3* mRNA and protein levels, but had no effect on levels of *NCX1* and *NCX2*, suggesting that different NCX isoforms might have diverse transcriptional mechanisms during OGD injury. The downregulation of *NCX3* mRNA levels might be a result of the biochemical aberrations that affect gene expression. Furthermore, we found that the expression levels of NCX3 protein were lower than that of mRNA. The decline of the NCX3 protein may be due to the downregulation of *NCX3* mRNA and the cleavage of the NCX3 protein by proteolytic enzymes activated under ischemia, such as caspases and calpain [36,37]. Catalpol treatment substantially increased the expression of *NCX3* mRNA and protein in PreOLs after OGD. A possible mechanism that accounts for these effects of catalpol is its antioxidant mechanism, which is crucial for maintaining intracellular homeostasis under ischemia. Intracellular homeostasis of PreOLs mediated by catalpol might prevent reduced synthesis of mRNA and increased degradation of NCX3 protein. However, additional studies are needed to determine the direct effects of catalpol on NCX3. Inhibition of NCX3 activity with NCX3-preferring inhibitor KB-R7943 increased the intracellular Ca^2+^ concentration and reversed catalpol-mediated cytoprotection. These findings indicate that NCX3 is the main contributor to NCX activity in PreOLs and operates in a forward mode during OGD. Reduced NCX3 function is involved in Ca^2+^-mediated PreOL damage or death under OGD. The protective effects of catalpol on PreOLs might be associated with the increased activity of NCX3, which facilitates intracellular Ca^2+^ extrusion.

Early white matter injury resulting from hypoxia–ischemia in preterm infants usually leads to adverse neurological outcomes such as mental retardation and cerebral palsy [38]. In the current study, neonatal hypoxia–ischemia in rats at P2 was sufficient to induce motor and cognitive disturbances combined with dysmyelination for 4 weeks post injury, and these effects were attenuated by catalpol. Hypomyelination might result from PreOL loss due to increased apoptosis, as supported by our in vivo and in vitro experiments. Hypomyelination may also be the result of arrested PreOL differentiation. Several studies have found that early OPCs that are resistant to hypoxia–ischemia proliferate robustly after PreOL degeneration and differentiate into PreOLs in acute white matter injury [39,40]. However, these newly generated PreOLs display persistent differentiation arrest in chronic white matter injury, which may adversely influence myelination and subsequent white matter maturation [41,42,43]. Hence, there is a need for therapies that promote PreOL maturation and enhance myelination during chronic white matter injury. Catalpol promoted PreOL maturation after neonatal hypoxia–ischemia or OGD injury, as supported by the increased MBP expression. These beneficial effects of catalpol may be achieved by preventing PreOL death in acute lesions and promoting newly generated PreOL differentiation in chronic lesions. It has also been reported that blockade of NCX with KB-R7943 decreases MBP synthesis [44]. Knocking out or silencing *NCX3*, but not *NCX1* and *NCX2*, impairs OPC differentiation [15]. Consistent with previous findings, our data showed that KB-R7943 can reverse the effect of catalpol on MBP expression in PreOLs after OGD. This result supports that catalpol promotes PreOL maturation by improving NCX3 function during ischemia.

ERK1/2 signaling is activated during oxidative stress and plays important roles in cell survival/death responses, depending on the cell type and stimulus duration [45,46]. Dual roles of ERK1/2 as a promoter of cell survival/death have also been reported in OLs. Delayed and sustained ERK1/2 activation under ischemic stimulation leads to OL death [47], whereas rapid and transient ERK activation after AMPA receptor stimulation promotes OL survival [7,29]. In this study, the level of ERK1/2 phosphorylation and the number of PreOL death were significantly increased after OGD, indicating that ERK1/2 activation induces PreOL death. Catalpol inhibited the activation of ERK1/2 under OGD, suggesting that the ERK1/2 signaling pathway may be involved in the protective effects of catalpol on PreOLs subjected to ischemia. Furthermore, we found that the inhibitory effects of catalpol on ERK1/2 activation were significantly reversed by applying KB-R7943. These results demonstrate that ERK1/2 activation is dependent on NCX3 dysfunction and intracellular Ca^2+^ overload. ERK1/2 activation might be an upstream and downstream event of mitochondrial damage in PreOLs after intracellular Ca^2+^ overload. The mitochondrion is an important organelle for Ca^2+^ buffering in OLs [29]. Excessive mitochondrial Ca^2+^ uptake contributes to ROS generation and ERK1/2 activation. This activated ERK1/2 might further aggravate mitochondrial damage, ultimately leading to cell death.

PARP-1 is an ERK1/2 target that can be activated by phosphorylated ERK1/2 through a direct interaction that is not related to DNA binding or DNA damage [48]. PARP-1 is crucial for cell death during ischemic cerebral injury through a process involving the depletion of NAD^+^, resultant energy failure, and mitochondrial dysfunction [49]. Previous studies have demonstrated that ERK1/2-mediated PARP-1 activation contributes to OL death under ischemic-reoxygenation damage [46]. PARP-1 activation also leads to the degeneration of OL processes in experimental models of multiple sclerosis [50,51]. The inhibition of PARP-1 activation by *PARP-1* gene depletion or pharmacological inhibitors can prevent OL damage and myelin depletion in mouse models of periventricular leukomalacia [49]. In parallel with these studies, our results revealed that OGD-induced PARP-1 activation was dramatically inhibited by catalpol and that this effect was markedly blocked by KB-R7943. These observations suggest that PARP-1 activation occurs downstream of NCX3 dysfunction and that PARP-1 is involved in catalpol-mediated cytoprotection for PreOL survival and maturation.

In summary, we have demonstrated that catalpol promotes PreOL survival and improves myelination and behavioral functions in a rat model of neonatal hypoxia–ischemia. Moreover, catalpol also suppressed PreOL death and differentiation arrest in an in vitro ischemia model through regulation of intracellular Ca^2+^ homeostasis and inhibition of mitochondrial damage, followed by attenuation of ERK1/2 and PARP-1 activation. These beneficial effects of catalpol might be related to the upregulation of NCX3. The present study indicates that catalpol could be a promising agent to prevent or treat white matter damage.

## 4. Materials and Methods

### 4.1. Neonatal Hypoxia–Ischemia Model and Drug Administration

Sprague-Dawley (SD) rats were obtained from the Experimental Animal Center of Army Medical University. All animal experiments were approved by the Laboratory Animal Welfare and Ethics Committee of Army Medical University (License number: SYXK-PLA-20120031, 1 July 2016). Postnatal day 2 (P2) SD rats were housed with their mothers and randomly allocated to the following three groups: sham-control group (Sha), vehicle-treated group (Veh), and catalpol-treated group (Cat). The neonatal hypoxia–ischemia model was induced by a bilateral common carotid ligation followed by hypoxia as previously described [52]. Briefly, rats of both sexes were lightly anesthetized with 2.5% halothane. The bilateral common carotid arteries were isolated and permanently ligated. At the end of a 30-min recovery period, rat pups were exposed to hypoxia (8% O_2_/92% N_2_) at 37 °C for 10 min. Then, the animals were returned to their dams. Rats in the Cat and Veh groups received catalpol (purity > 98%, National Institute for the Control of Pharmaceutical and Biological Products, Beijing, China) (5 mg/kg, dissolved in physiological saline) or physiological saline alone administered intraperitoneally immediately after hypoxia exposure and then every 24 h for 5 days. The Sha group received the same operation without ligation and hypoxia.

### 4.2. Rotarod Test

The Rotarod test was used to evaluate the motor coordination and balance of rats [25]. On P22, the test was preceded by habituation and each animal had three trials within 5 min between each trial. Rats were placed on a stationary cylinder (Biowill, Shanghai, China) for 30 s and thereafter for 2 min with a constant-speed rotation of 5 revolutions per minute (rpm). Rats that fell from the cylinder were placed on it again until they were able to stay for 60 s. From P23 to P25, rats were placed on the rotating cylinder at a speed of 5 rpm that was gradually accelerated to 40 rpm in 5 min during the test. If the rat fell from the cylinder or remained consecutively on the cylinder for two cycles, the test was stopped. The total time the rat remained on the cylinder was recorded. After a 15-min rest, the test was repeated. Each rat was tested on the Rotarod three times per day for 3 consecutive days. The average latency of 3 days was calculated and used for analysis.

### 4.3. Morris Water Maze Test

Rat spatial learning and memory abilities were evaluated with the Morris water maze test [26]. The Morris water maze (Biowill) was filled with water of 23 ± 0.5 °C. The circular pool was divided into four quadrants (north, south, east, and west locations), and the platform was always placed in a fixed location and submerged under opaque water. From P26 to P29, rats received four training trials per day with a 20-min inter-trial interval for 4 consecutive days. During the training, rats were randomly placed in the water in the four quadrants of the pool. Each rat was given 60 s to found the hidden platform and allowed to remain on it for 15 s. After staying on the platform, the rat was placed into a heated cage for the next trial. The rats that failed to find the platform within 60 s were guided onto the platform and allowed to remain there for 30 s to enhance their spatial memory. The escape latency (time to reach the platform) was recorded every time. On the 5th day (at P30), the platform was removed and the rats were put in the water in the opposite quadrant to perform a 60-s probe test. The number of crossings of the previous platform location was recorded in the probe test.

### 4.4. Immunohistochemistry

On P6 and P30, rat pups were anesthetized and perfused transcardially with saline followed by 4% paraformaldehyde. The coronal brain blocks including the corpus callosum were dissected, and free-floating sections (20 µm) of the brain were sliced in 4% paraformaldehyde at 4 °C. The free-floating sections were incubated in 3% H_2_O_2_ to suppress endogenous peroxidase activity. After blocking in PBS containing 5% bovine serum albumin (BSA) and 0.4% Triton X-100 for 30 min at 37 °C, the sections were incubated with mouse anti-O4 (1:100, Sigma-Aldrich, St. Louis, MO, USA) antibody and goat anti-myelin basic protein (MBP) (1:200, Santa Cruz Biotechnology, San Francisco, CA, USA) overnight at 4 °C. The sections were then washed again with PBS and incubated with appropriate horseradish peroxidase-conjugated secondary antibodies (1:200, Santa Cruz) at 37 °C for 3 h. Finally, the immunoreaction products of the sections were visualized with a DAB kit (Zhongshan, Beijing, China) [23]. Negative controls were set up by omitting the primary antibodies.

### 4.5. Oligodendrocyte Progenitor Cell Culture.

Rat oligodendrocyte progenitor cells (OPCs) were propagated by established methods [53]. Briefly, mixed glial cells were prepared from the cerebral cortices of neonatal (P1–P3) SD rat pups and cultured in DMEM/F12 containing 10% fetal bovine serum for 5 days. OPCs were enriched through two passages in OPC-proliferation medium (DMEM/F12 supplemented with 15% B104-conditioned medium and 1% N2-supplement). For the indicated experiments, the purified OPCs were plated onto poly-d-lysine-coated cover slips or dishes. After 12 h of recovery in OPC-proliferation medium, OPCs were initiated to differentiate by cultivating the cells in OPC-differentiation medium (DMEM/F12 supplemented with 1% N2 supplement, 1% fetal bovine serum, and 5 mg/mL insulin). The purity of the OPC cultures was monitored by detecting cell morphology and was identified by immunostaining with cell type-specific markers; only cultures with >95% purity were used for experiments. On day 3 of differentiation, >90% of the cells were identified as O4^+^ PreOLs. The cells were used for various experiments at the indicated time points.

### 4.6. OGD Model and Drug Treatment

PreOLs were divided into four groups as follows: control group (CTL), OGD-treated group (OGD), catalpol-treated group (CAT), and KB-R7943+catalpol-treated group (KB+CAT). The OGD model was established as described previously to mimic an in vitro model of cerebral ischemia [24]. Briefly, PreOL cultures were incubated in glucose-free DMEM medium (Gibco Life Technologies, Carlsbad, CA, USA) with 8 mM Na_2_S_2_O_4_ (Sigma-Aldrich, St Louis, MO, USA) at 37 °C for 30 min to scavenge O_2_ molecules in solution and to reduce the partial pressure of O_2_ to zero. Following OGD, the cells were maintained in glucose-containing medium in a 5% CO_2_-containing atmosphere at 37 °C for an additional 12 h. Cells in the CAT group were pretreated with 0.5 mM catalpol for 1 h prior to OGD. Cells in the KB+CAT group were simultaneously pretreated with 10 μM NCX inhibitor KB-R7943 (Sigma-Aldrich) and 0.5 mM catalpol for 1 h prior to OGD. The CTL group was maintained under a normoxic atmosphere in glucose-containing medium without catalpol or KB-R7943 treatment.

### 4.7. MTT Assay

Cell viability was measured using 3-(4,5-dimethylthiazol-2-yl)-2,5-diphenyltetrazolium bromide (MTT) assays according to the manufacturer’s instructions (Beyotime, Haimen, Jiangsu, China) [54]. Briefly, cells were harvested and seeded in a 96-well plate (100 μL/well). After OGD treatment, 10 μL of MTT solution (5 mg/mL) was added to each well and incubated for an additional 4 h at 37 °C. After removal of the supernatants, 100 μL of formazan lysis solution was added to each well to dissolve to the MTT-formazan. The absorbance was measured with a microplate reader (Bio-Rad, Hercules, CA, USA) at a wavelength of 570 nm.

### 4.8. Lactate Dehydrogenase Assay 

The release of cytosolic lactate dehydrogenase (LDH), an indicator of cytotoxicity and plasma membrane damage, was determined by using a commercially available kit (Beyotime) according to the manufacturer’s protocol [55]. At the indicated time points, the culture supernatant was collected and transferred to a 96-well plate. The cells were solubilized with lysis buffer, and the supernatant was used for LDH detection. The supernatant was incubated with LDH working solution in the dark at room temperature for 30 min. The release of cytosolic LDH to the medium was read at 490 nm and was calculated as the percentage of LDH in the culture medium versus total LDH activity (LDH released into medium + LDH released from lysed cells).

### 4.9. Terminal Deoxynucleotidyl Transferase-Mediated Dutp-Biotin Nick End Labeling Assay (TUNEL) Staining

TUNEL staining was performed by using an In Situ Cell Death Detection Kit (Roche, Mannheim, Germany) in accordance with the manufacturer’s instructions [24]. After rinsing with PBS, the cells were treated with 3% H_2_O_2_ in methanol for 10 min at room temperature. The cells were then permeabilized with a solution containing 0.1% Triton X-100 and 0.1% sodium citrate for 2 min on ice. Thereafter, the cells were incubated with the TUNEL reaction mixture for 1 h at 37 °C in the dark and washed twice with PBS. Finally, the cells were counterstained with 4′,6′-diamidino-2-phenylindole (DAPI) (0.1 μg/mL in PBS, Sigma-Aldrich) to visualize nuclei. Negative controls were prepared using Label Solution instead of the TUNEL reaction mixture.

### 4.10. Immunofluorescent Staining

Cells cultured in 24-well plates (4.5 × 10^4^ cells/well) were fixed with ice-cold methanol for 20 min at −20 °C, followed by PBS washes. After blocking in PBS containing 5% BSA and 0.4% Triton X-100 for 30 min at 37 °C, the cells were incubated with goat anti-MBP antibody (1:200, Santa Cruz) and rabbit anti-NCX3 antibody (1:200, Santa Cruz) overnight at 4 °C. The cells were then washed again with PBS and incubated with Alexa Fluor 568/488-coupled secondary antibody (1:1000, Invitrogen, Carlsbad, CA, USA) for 2 h at room temperature. After washing, the cells were counterstained with DAPI for 5 min to visualize all nuclei. Finally, the cells were sealed and images were acquired using a laser scanning confocal microscope (Olympus IV1000, Tokyo, Japan) [24]. The optical density of MBP and NCX3 staining was analyzed with the Image J program (National Institutes of Health, Bethesda, MD, USA). The fluorescent images were first converted to grayscale images in a white background. After setting threshold levels, the mean gray value of selected area was measured. The mean gray value represents the optical density.

### 4.11. Confocal Ca^2+^ Imaging

The intracellular Ca^2+^ at the resting condition was detected by fluorescent probe Fluo-3/AM (Beyotime) according to our previous study [24]. Briefly, cells grown in glass-bottomed dishes were first washed with PBS and then loaded with 5 μM Fluo-3/AM (Beyotime) in assay buffer solution (135 mM NaCl, 2 mM glucose, 8 mM HEPES, 2 mM MgCl_2_, 3 mM KCl, and 2.2 mM CaCl_2_; pH 7.4) for 20 min at 37 °C. The loading solution was washed out with PBS and the cells were incubated for at least 20 min at room temperature to allow complete dye de-esterification. Real-time intracellular Ca^2+^ response were monitored under 30 mM high-glucose stimulation. Fluorescence images were acquired and measured by a confocal laser scanning microscope (Olympus IV1000) (488 nm excitation and 525 nm emission wavelengths) and Fluoview image processing software (Olympus v2.1). The Ca^2+^ fluorescence intensity was quantified using the Image J program (National Institutes of Health). Independent experiments were performed six times with 15–30 cells recorded for each experiment.

### 4.12. Quantitative RT-PCR

Total RNA was isolated from cultured cells using TRIzol reagent (Life Technology, Carlsbad, CA, USA) and its quality was confirmed by spectrophotometry and agarose gel electrophoresis. The extracted RNA was reverse transcribed to cDNA using a PrimeScript™ RT-PCR Kit (Takara, Tokyo, Japan) according to the manufacturer’s instructions. The cDNA was tested by real-time PCR with a Rotor Gene6000 (Corbertt Research, Sydney, Australia) according to the manufacturer’s protocols and was analyzed by the 2^−ΔΔ^*^C^*^t^ method. Briefly, PCR analyses were performed using SYBR premix Ex Taq (Takara) in a final reaction volume of 20 μL. The primers used for expression detection were as follows: *NCX1* (5′-CTGGAGCGCGAGGAAATGTTA-3′ and 5′-GACGGGGTTCTCCAATCT-3′); *NCX2* (5′-AGGAGGCCGCACACCTTTCC-3′ and 5′-CAAGGCGTGGCTGGGCTCTC-3′); *NCX3* (5′-GGCTGCACCATTGGTCTCA-3′ and 5′-GACGGGGTTCTCCAATCT-3′); and *β-actin* (5′-CATCTCTTGCTCGAA-GTCCA-3′ and 5′-ATCATGTT-TGAGACCTTCAACA-3′) [17]. An Image-Pro Plus image analysis system was used to analyze the optical density of the PCR product bands, which were normalized to those of *β-actin*.

### 4.13. Western Blot Analysis

After washing with PBS, cells seeded in 60-mm dishes (3.0 × 10^5^ cells/mL) were centrifuged at 12,000 *g* for 30 min at 4 °C. Total cells were solubilized in ice-cold RIPA lysis buffer (Millipore, Billerica, MA, USA) for 30 min, and protein concentrations were determined by the Bradford method. Following boiling for 5 min in loading buffer, protein cell lysates were separated by SDS-PAGE, followed by transfer of the proteins to polyvinylidene difluoride membranes (Millipore) and blocking with 1% nonfat dried milk in 10 mM PBS with 0.05% Tween-20 (PBST) for 2 h. The membranes were incubated overnight at 4 °C with the following primary antibodies: MBP (1:500, Santa Cruz), p-ERK1/2 (1:500, Cell Signaling Technology, Beverly, MA, USA), ERK1/2 (1:500, Cell Signaling), activated poly-ADP-ribose polymerase-1 (PARP-1) (1:500, Santa Cruz), and β-actin (1:2000, Santa Cruz). The membranes were washed with PBST and incubated with peroxidase-conjugated secondary antibodies (1:5000, Santa Cruz) at 37 °C for 3 h. Protein bands were visualized by chemiluminescence (ECL kit, Amersham, Piscataway, NJ, UK) [24].

### 4.14. Mitochondrial Membrane Potential Assay 

Mitochondrial membrane potential was measured using a JC-1 probe (Beyotime) according to the manufacturer’s directions. After treatment, cells grown in 24-well plates were incubated with JC-1 staining solution (5 μg/mL) for 20 min at 37 °C and washed twice with JC-1 staining buffer [56]. JC-1 aggregates emit red fluorescence in intact mitochondria (excitation wavelength of 585 nm and emission wavelength of 590 nm), whereas monomers emit green fluorescence in depolarized mitochondria (excitation wavelength of 514 nm and emission wavelength of 529 nm). Fluorescent images were acquired using a fluorescence microscope (Olympus IV1000). The fluorescence intensity was analyzed with the Image J program. The mean gray value represents the intensity of fluorescence. The loss of mitochondrial membrane potential is indicated by a decrease in the red/green fluorescence intensity ratio.

### 4.15. Determination of Reactive Oxygen Species (ROS) Generation

Intracellular reactive oxygen species (ROS) levels were investigated using an ROS assay kit (Beyotime). Briefly, cells grown in 60-mm dishes were harvested and incubated with 10 μM dichlorodihydrofluorescein diacetate (DCFH-DA) at 37 °C for 20 min. DCFH-DA was oxidized by ROS to form the fluorescent product DCF. After washing three times with serum-free culture medium, the fluorescence intensity of DCF was measured at 488 nm for excitation and 525 nm for emission with a fluorescent plate reader (Bio-TEK, Winooski, VT, USA) [57].

### 4.16. Determination of Malondialdehyde Levels

Cells were harvested and homogenized in ice-cold RIPA lysis buffer (Millipore), and the lysates were centrifuged at 1600× *g* for 10 min at 4 °C. The supernatants were collected for protein concentration determination. The levels of malondialdehyde (MDA) were detected using a Lipid Peroxidation MDA assay kit (Beyotime) according to the manufacturer’s instructions [58]. The absorbance of the reaction products was detected with a microplate reader (Bio-Rad) at a wavelength of 532 nm.

### 4.17. Statistical Analysis

All data are presented as the mean ± SEM. One-way analysis of variance was performed, followed by Tukey’s multiple comparison tests. All statistical analyses were performed using SPSS 12.0 (SPSS Inc., Chicago, IL, USA) for Windows. Values of *p* < 0.05 were considered statistically significant.

## Supplementary Materials

Supplementary materials can be found at http://www.mdpi.com/1422-0067/19/7/1925/s1.

## Abbreviations

PreOL	Premyelinating oligodendrocyte
OL	Oligodendrocyte
OPC	Oligodendrocyte progenitor cell
NCX3	Na^+^/Ca^2+^ exchanger 3
OGD	Oxygen-glucose deprivation
ERK1/2	Extracellular signal-regulated kinase 1/2
PARP-1	Poly-ADP-ribose polymerase-1
KB-R7943	2-[2-[4-(4-nitrobenzyloxy)phenyl]ethyl]isothiourea
MBP	Myelin basic protein
ROS	Reactive oxygen species
MDA	Malondialdehyde
