# Supplementary material for: Catalpol Inhibits Ischemia-Induced Premyelinating Oligodendrocyte Damage through Regulation of Intercellular Calcium Homeostasis via Na+/Ca2+ Exchanger 3
Source: Int J Mol Sci. 2018 Jun 30;19(7):1925. doi: 10.3390/ijms19071925 (PMC6073132; doi:10.3390/ijms19071925)
Supplement: Supplementary file 1 [file ijms-19-01925-s001.pdf]

Supplementary materials: Catalpol Inhibits Ischemia-Induced Premyelinating Oligodendrocyte Damage through Regulation of Intercellular Calcium Homeostasis via Na^+/Ca^{2+} Exchanger 3

Qiyan Cai, Teng Ma, Yanping Tian, Chengren Li and Hongli Li *

Chongqing Key Laboratory of Neurobiology, Department of Histology and Embryology, College of Basic Medicine, Army Medical University (Third Military Medical University), Chongqing 400038, China; fengcai1112@126.com (Q.C.); matt0119@163.com (T.M.); tianyp1981@163.com (Y.T.); lichengren@sohu.com (C.L.)

* Correspondence: lihongli@tmmu.edu.cn; Tel.: +86-23-6875-2220.

Received: 12 May 2018; Accepted: 22 June 2018; Published: date

Supplementary materials and methods

Drug treatment and Ca^{2+} imaging

Cultured PreOLs were divided into five groups as follows: control group (CTL), OGD-treated group (OGD), catalpol-treated group (CAT), KB-R7943+catalpol-treated group (KB+CAT), and NCX3 antibody+catalpol-treated group (Antibody+CAT). PreOL cultures in the OGD group were incubated in glucose-free DMEM medium (Gibco) with 8 mM $Na_2S_2O_4$ (Sigma) at 37°C for 30 min to scavenge O_2 molecules in solution and to reduce the partial pressure of O_2 to zero. Following OGD, the cells were maintained in glucose-containing medium in a 5% CO_2-containing atmosphere at 37°C for an additional 12 h. Cells in the CAT group were pretreated with 0.5 mM catalpol for 1 h prior to OGD. Cells in the KB+CAT group were simultaneously pretreated with 10 μM NCX inhibitor KB-R7943 (Sigma*)* and 0.5 mM catalpol for 1 h prior to OGD. Cells in the Antibody+CAT group were simultaneously pretreated with 2.5 μg/ml NCX3 antibody (Santa Cruz) and 0.5 mM catalpol for 1 h prior to OGD. The CTL group was maintained under a normoxic atmosphere in glucose-containing medium without catalpol, KB-R7943, or NCX3 antibody treatment. Real-time intracellular Ca^{2+} response under 30 mM high-glucose stimulation were monitored by a confocal laser scanning microscope (Olympus). Independent experiments were performed six times with 15-30 cells recorded for each experiment.

Supplementary results

KB-R7943 and NCX3 antibody have similar effects on intracellular Ca^{2+} response under OGD in presence of catalpol

In the CTL group, intracellular Ca^{2+} concentrations showed a transient elevation, and then rapidly returned to basal levels following high-glucose stimulation. By contrast, intracellular Ca^{2+} concentrations rose steadily throughout the period in PreOLs subjected to OGD. The total volume of intracellular Ca^{2+} in the OGD group was strongly increased, as shown by the elevated area under the curve compared to the CTL group ($P < 0.01$). However, catalpol treatment improved the recovery of Ca^{2+} concentrations toward to basal levels under high-glucose stimulation. The total volume of intracellular Ca^{2+} was significantly decreased following catalpol treatment, as demonstrated by the decreased area under the curve compared with the OGD group ($P < 0.05$). In the KB+CAT and Antibody+CAT groups, intracellular Ca^{2+} concentrations rose rapidly, and then decayed to a higher plateau after high-glucose stimulation. The total volume of intracellular Ca^{2+} was significantly elevated in the KB+CAT group ($P < 0.05$) and Antibody+CAT group ($P < 0.05$), as indicated by the elevated area under the curve relative to the CAT group. However, there was no significant difference between the KB+CAT group and Antibody+CAT group in the total volume of intracellular Ca^{2+} under OGD and high-glucose stimulation. These data demonstrated that KB-R7943 and NCX3 antibody have similar effects on intracellular Ca^{2+} response under OGD in presence of catalpol (Figure S1).

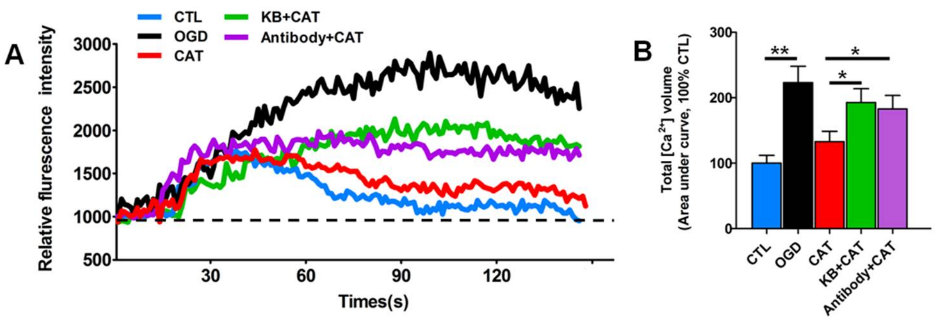

Figure S1. Effects of KB-R7943 and NCX3 antibody on intracellular Ca^{2+} response under OGD in presence of catalpol. (A) Representative Ca^{2+} response traces after high-glucose stimulation in the CTL, OGD, CAT, KB+CAT, and Antibody+CAT groups. **(B)** Quantification of total volume of intracellular Ca^{2+} under high-glucose stimulation in the CTL, OGD, CAT, KB+CAT, and Antibody+CAT groups. The area under the curve represents the total volume of intracellular Ca^{2+}. Six separate experiments were conducted and 15-30 cells were recorded for each experiment. Data are shown as means ± SEM. $*p < 0.05$; $**p < 0.01$.
